# Exploring the role of orbital exenteration in survival among patients with severe rhino-orbital cerebral mucormycosis: a case series

**DOI:** 10.3389/fopht.2025.1689076

**Published:** 2025-12-02

**Authors:** Shaili S. Davuluru, Andrew J. Clark, Kenneth Cahill, Jill A. Foster, Sandy Zhang-Nunes

**Affiliations:** 1 Roski Eye Institute, Department of Ophthalmology, Keck School of Medicine, University of Southern California, Los Angeles, CA, United States; 2 Central Ohio Eye and Plastic Surgery, Nationwide Children’s Hospital, Department of Ophthalmology, The Ohio State University, Columbus, OH, United States; 3 Kaiser Permanente Los Angeles Medical Center, Southern California Permanente Medical Group, Los Angeles, CA, United States

**Keywords:** orbital exenteration, mucormycosis, rhino-orbital, invasive fungal sinusitis, globe-sparing, retrobulbar amphotericin B

## Abstract

Rhino-orbital cerebral mucormycosis (ROCM) is a rare, but potentially fatal opportunistic infection due to a fungus of the Mucorales sp. that involves infiltration of the sinuses, orbits, and central nervous system. Orbital exenteration (OE) is reserved for severe cases refractory to initial antifungal treatment and endoscopic sinus debridement, though there is currently no consensus on the ideal timing of OE and its impact on infection progression and survival. In this case series, we present three cases of similar, extensive ROCM that were candidates for OE after initial medical and surgical management with variable outcomes. We discuss that undergoing OE does not necessarily correlate with survival after ROCM infection and that the decision to proceed with exenteration should be personalized based on the status of a patient's comorbidities and the nature and extent of infectious spread. Globe-sparing OE may serve as a viable alternative for patients with localized, late-stage infection or reversible ROCM risk factors given vision preservation and less postoperative disfigurement with possibly comparable efficacy.

## 1 Introduction

Rhino-orbital cerebral mucormycosis (ROCM) is a rare, but potentially fatal infection due to fungus of the Mucorales order that invades the paranasal sinuses, orbits, and central nervous system (CNS). Mucormycosis predominantly affects the immunocompromised population, most commonly those with uncontrolled diabetes (1, 2). The global incidence rate of ROCM ranges from 0.005 to 1.7 cases per 1 million population with a marked uprise in ROCM incidence globally since the COVID-19 pandemic (2–6).

Early diagnosis and treatment of ROCM is a critical factor for improved outcomes and survival (7, 8). Patients with ROCM infection may initially present with non-specific symptoms such as headache, fever, nasal discharge, and sinusitis. The infection can progress rapidly, resulting in periorbital edema, proptosis, ophthalmoplegia, or even loss of vision. Management begins with the reversal of the underlying immunosuppression, systemic antifungal medication (e.g., amphotericin and micafungin), and endoscopic sinus surgery (ESS) for debridement of necrotic tissue (9). Antifungals can also be administered as intraoperative irrigation, infraorbital infusion through surgically placed drain, or retrobulbar injection for moderate to severe cases (9). Orbital exenteration (OE) is reserved for significant orbital involvement refractory to all treatment methods as a life-salvage measure. Conventional OE involves removal of all orbital contents including the entire globe and surrounding structures such as extraocular muscles, periorbital fat, nerves, and vasculature. Modified forms of exenteration may preserve some of these orbital structures such as the globe, lid, and/or conjunctiva. This may pose a less disfiguring alternative that preserves some function and has similar survival outcomes. The benefit, ideal timing, type of OE, and its impact on infection progression and survival continue to be debated (4–15). In this case series, we describe three cases of similar severe ROCM presentation, with orbital symptoms including proptosis, edema, and/or restricted extraocular movements (EOMs), that had different surgical interventions and outcomes. Our intention is to explore and review the role and impact of OE in eliminating late-stage infection and patient survival.

## 2 Case descriptions

Key components of history, exam findings, and management of all three cases are summarized in **Table 1**.

**Table 1:** Summary of three ROCM cases.

Case	Background		Exam findings				Treatment history			Outcome
	Age, gender	Comorbid conditions	Initial	OE decision	Final	CNS involved?	Amphotericin B?	ESS?	OE?	
1	54, Female	Alcoholic cirrhosis DM (A1c 13.9) with DKA	BCVA OD: 20/30+ Pupils: Brisk, no RAPD EOMs: Restricted OD	BCVA OD: 20/50 Pupils: Brisk, no RAPD EOMs: Restricted OD (stable)	BCVA OD: 20/25 Pupils: Brisk, no RAPD EOMs: Restricted in up/downgaze OD (improved)	Yes	Intraorbital AmpB 5 mL (1 mg/mL) ×4 days (via JP drain) Intravenous AmpB 650 mg daily*	Yes, ×3	No, deferred	Alive, successful reconstructions
2	81, Female	DM, ESRD Breast cancer with active CTX	BCVA OD: 20/200- OS: 20/400 (stable) Pupils: Slow, no RAPD EOMs: Restricted OS > OD in up/downgaze and abduction	BCVA OD: 20/400 OS: 20/800 Pupils: Slow, 1+RAPD EOMs: Restricted OS > OD in up/downgaze and abduction (worse)	NA	Yes	Intravenous AmpB 10 mg/kg daily	Yes, ×3	Yes, complete OE	Deceased
3	8, Male	Acute myeloid leukemia with CTX	BCVA OS: 20/25- Pupils: Brisk, no RAPD EOMs: Restricted OS in up/downgaze, abduction	BCVA OS: 20/25- Pupils: Brisk, no RAPD EOMs: Restricted OS (stable)	BCVA OS: 20/20 Pupils: Brisk, no RAPD EOMs: Restricted OS (improved)	No	Intraorbital AmpB ×1 (intra-operative) Intravenous AmpB daily	Yes	Yes, globe-sparing medial OE	Alive, successful reconstructions

BCVA, best-corrected visual acuity; RAPD, relative afferent pupillary defect; EOM, extraocular movement; CNS, central nervous system; DM, diabetes mellitus; DKA, diabetic ketoacidosis; ESRD, end-stage renal disease; CTX, chemotherapy; OE, orbital exenteration; AmpB, liposomal amphotericin B; ESS, endoscopic sinus surgery.

### 2.1 Case 1

**Figure 1:**
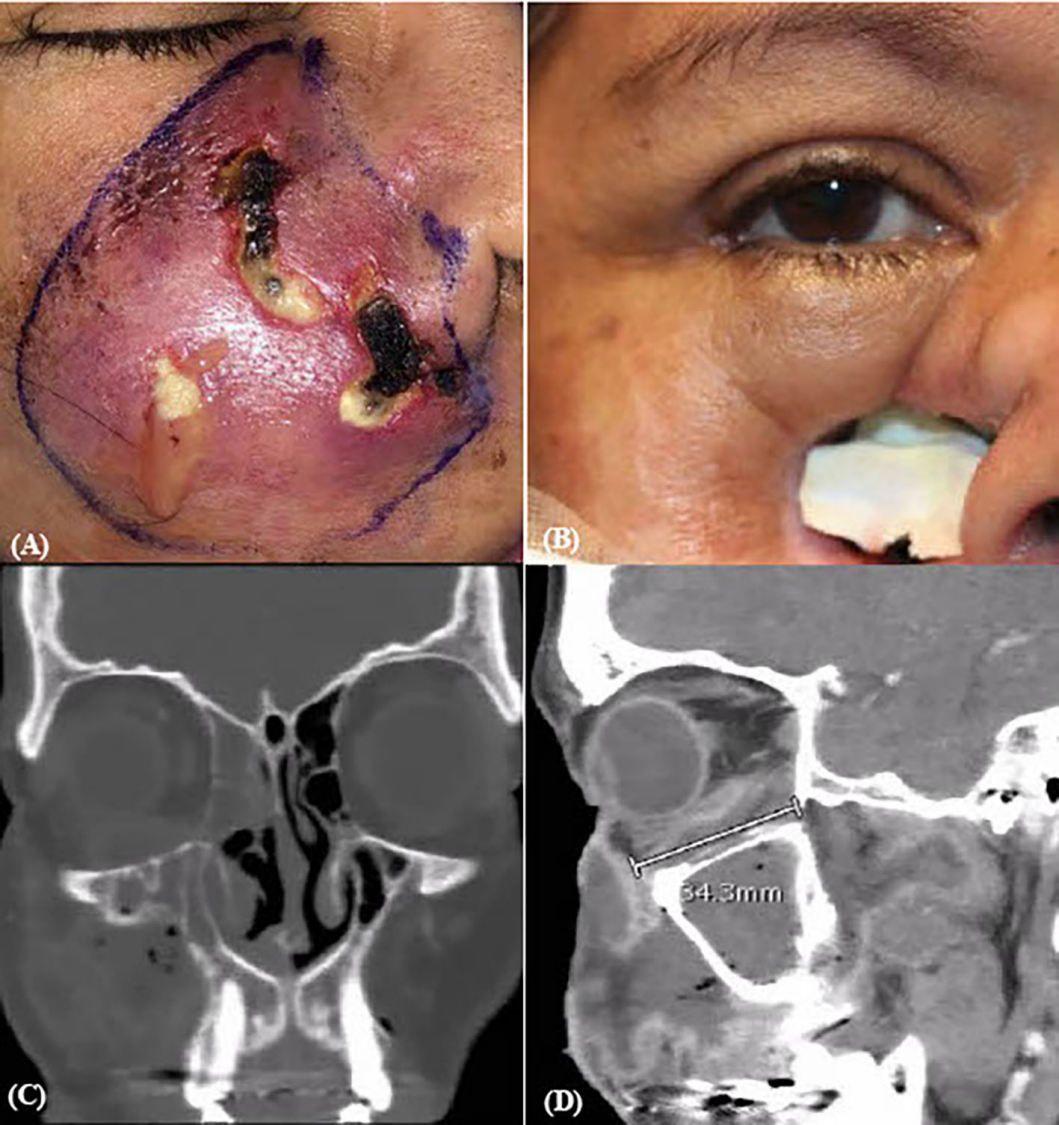
Pre- and post-operative surgical and CT imaging of case 1. **(A)** Pre-op ESS and **(B)** two months post-discharge gross images. CT orbit coronal **(C)** and sagittal **(D)** sections revealing continued mucormycosis extension into middle cranial fossa and adjacent structures.

A 54-year-old woman with a history of alcohol-related cirrhosis presented with nearly 4 weeks of worsening right-sided facial swelling and purulence. She was treated with empiric antibiotic therapy for upper respiratory infection symptoms. The patient was found to have diabetic ketoacidosis and was newly diagnosed with type 2 diabetes with a hemoglobin A1c level of 13.9%. On presentation to our hospital, ocular exam was significant for edema, erythema, and multiple purulent erosions of the skin overlying the right maxillary sinus (**Figure 1A**). Her right eye visual acuity was 20/25- and pupils were reactive without relative afferent pupillary defect (RAPD). Restricted EOMs of the right eye were present on abduction, up and downgaze (**Table 1**). Frozen sections and final fungal culture confirmed *Mucor* sp. for which intravenous liposomal amphotericin B and micafungin were initiated. The patient then underwent functional ESS with extensive debridement. Magnetic resonance imaging (MRI) of the orbits revealed dural enhancement and ROCM extension through to the middle cranial fossa (**Figures 1C, D**), prompting two subsequent debridements and daily infraorbital amphotericin B infusions for 4 days. Visual acuity diminished to 20/50- with persistent restriction of extraocular motility in the right eye. Given continued ROCM progression and continued intracranial involvement, OE was suggested. The patient eventually deferred OE for additional debridements due to uncertain success and impact on quality of life. On hospital day (HD) 17, the patient was stable for discharge to a skilled nursing facility on long-term intravenous antifungals. Visual acuity and extraocular motility improved, although there remained residual restriction on up and downgaze. Her clinical picture continued to stabilize 2 months post-operatively (**Figure 1B**) and she successfully underwent facial reconstruction 13 months post-infection.

### 2.2 Case 2

**Figure 2:**
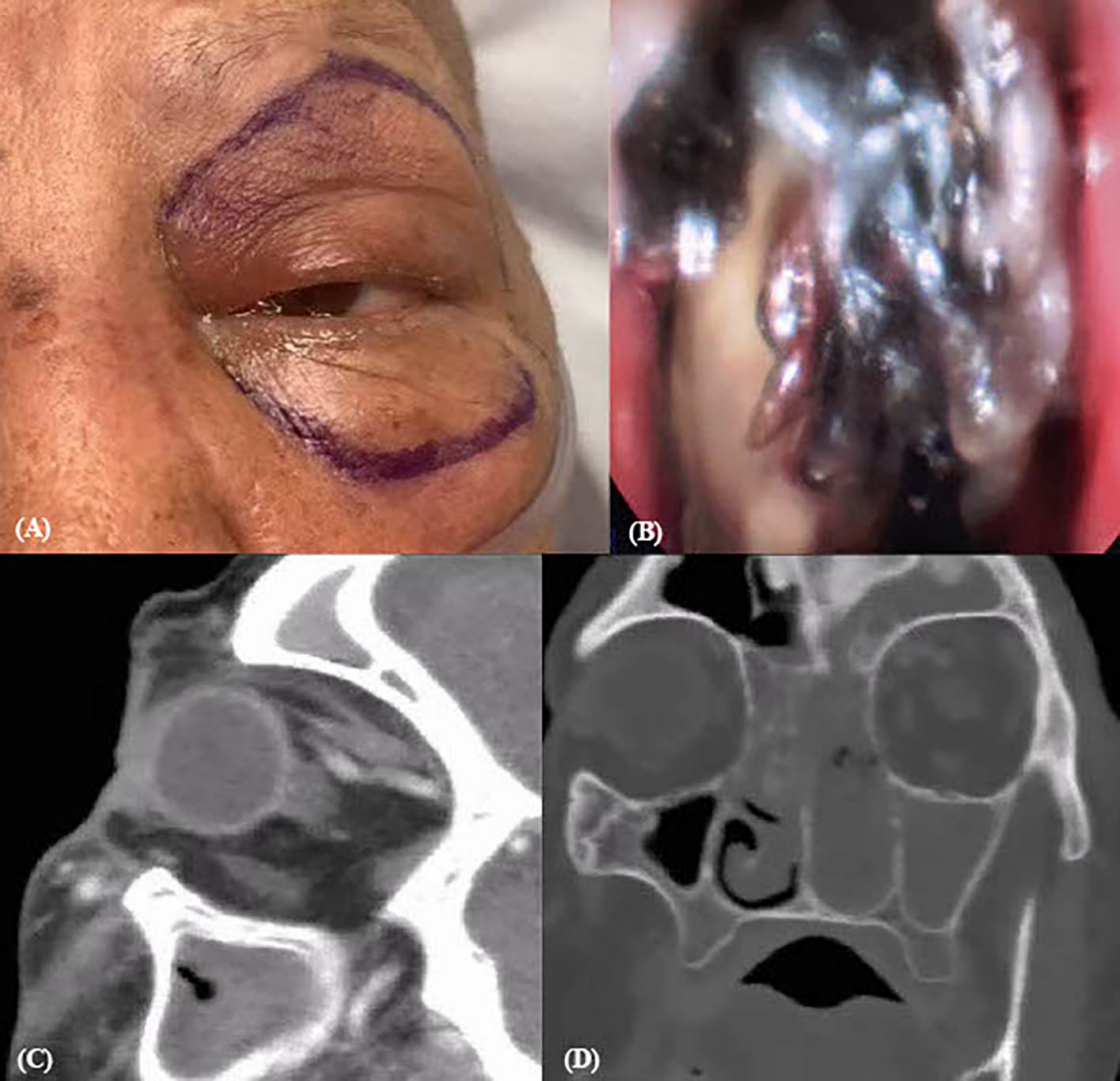
Pre-operative endoscopic and CT contrast imaging of case 2. **(A)** Pre-operative gross imaging. **(B)** Fungal growth on ESS. Sagittal **(C)** and coronal **(D)** view on CT orbit with contrast showing increased reticulation and stranding around left optic nerve and retro-orbitally.

An 81-year-old woman initially presented with 1 week of fever and edema of the left eye. She was found to have neutropenic fever with concern for pseudomonal pneumonia, leading to admission at an outside hospital. Pertinent medical history includes type 2 diabetes mellitus, end-stage renal disease, atrial septal defect and mitral valve regurgitation with pacemaker, and breast cancer for which the patient was undergoing chemotherapy treatment. Despite empiric antibiotics, she experienced worsening ocular symptoms with new-onset diplopia and proptosis (**Figure 2A**). This prompted transfer to our hospital for a higher level of care. On initial exam, best-corrected visual acuity was OD 20/200- and OS 20/400, with sluggish reactive pupils and no RAPD. The patient had restricted abduction, adduction, and inferior movement of the right eye greater than the left eye (**Table 1**). Left eye proptosis, mild periorbital edema and resistance to retropulsion, and left mucoid discharge with temporal chemosis were also noted.

Upon hospital admission, the patient started intravenous (IV) liposomal amphotericin B and micafungin. She then underwent ESS with extensive debridement during which necrotic tissue was visualized (**Figure 2B**). The resulting fungal culture was positive for *Rhizopus* sp. She continued antifungal treatment and received a second ESS without findings of new necrosis. However, 2 weeks after admission, the patient began to have worsening limitations of motility in all fields of gaze in the left eye greater than the right eye with a new left RAPD. Computed tomography (CT) of the orbits with contrast revealed increased reticulation and stranding around the optic nerve sheath and retro-orbital fat (**Figures 2C, D**). In subsequent days, MRI showed further progression with enhancement of intraconal and extraconal spaces extending to apex, indicative of orbital apex syndrome. As a result of persistent ROCM progression and signs of intracranial invasion as evidenced by ocular exam and imaging, the patient underwent OE and further debridement on HD18. After exenteration, the patient developed atrial fibrillation with rapid ventricular response leading to intensive care unit (ICU) admission. Her overall condition further deteriorated with respiratory decompensation due to multifocal pneumonia and multisystem organ failure from sepsis. After goals of care discussion and mutual decision-making with family, there was no further escalation of care. The patient was compassionately extubated on HD34.

### 2.3 Case 3

**Figure 3:**
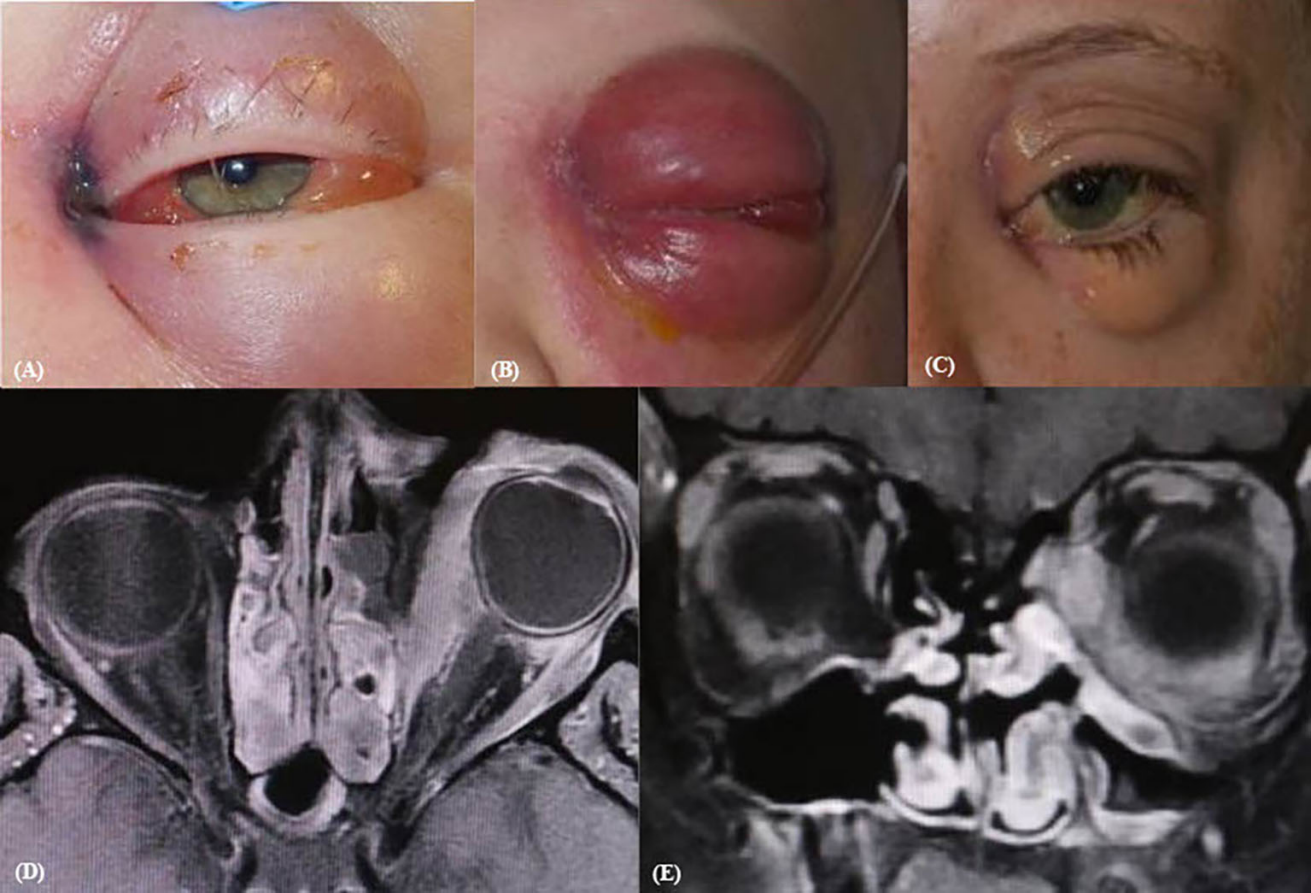
Pre- and post-operative imaging of case 3. Initial presentation pre-op **(A)**, clinical progression **(B)**, and post-reconstruction **(C)** gross images. Transverse **(D)** and coronal **(E)** CT orbit demonstrate visible peri-orbital enhancement and proptosis.

An 8-year-old male patient with a history of acute myeloid leukemia (AML) initially presented with subtle dark bruising of the left medial canthal region, which evolved into left medial orbital necrosis (**Figure 3C**) consistent with ROCM. On exam, left eye near visual acuity was 20/25- (J1-) with reactive pupils and no RAPD. Extraocular motility was notable for restriction on abduction, up and downgaze (**Table 1**). Intravenous amphotericin and micafungin were initiated. He received ESS with PCR positive for *Rhizopus* sp. His symptoms progressed with notable erythema, edema, and proptosis (**Figure 3D**) with persistent ophthalmoplegia. MRI of the orbits revealed a defined ischemic area limited to the medial orbit with preserved vascularity of the lateral orbit (**Figures 3A, B**), supporting the impression of ROCM progression. Of note, the patient’s AML was improving with expected immune reconstitution after a stem cell transplant. Given his improving immunity and the localized nature of ROCM, subtotal exenteration was advised. The patient then underwent globe-sparing medial OE with intraorbital amphotericin B irrigation intraoperatively. He had notable improvement of his clinical appearance with associated improvement of his visual acuity and motility (**Table 1**). He eventually underwent multiple successful reconstructions later in childhood (**Figure 3E**).

## 3 Discussion

Overall, mortality due to ROCM remains high, ranging up to 53% in cases with notable immunosuppressive risk factors and extensive disease causing loss of light perception (16–19). In a 2018 meta-analysis, Vaughan et al. found no improvement of the ROCM survival rate when compared to a previous review by Yohai et al. in 1994 (7, 8). The stable overall survival rate despite treatment developments over the years suggests that outcomes are likely determined by other clinical features, such as immunosuppressive risk factors and disease severity. This study and several others have identified poor prognosis after orbital mucormycosis in patients with features of older age, hemiparesis or hemiplegia, presence of facial necrosis, active COVID-19 infection, neutropenia, and/or delayed medical management, for instance (7, 8, 12). In a recent study with a 3-year follow-up, Zia et al. reported higher mortality in patients with diminished sensation in V2 distribution and absence of light perception at initial presentation, ultimately indicating implication of the orbit and the central nervous system (19).

There remains to be limited consensus on the benefit and timing of OE in cases of severe ROCM, with recent studies attempting to better understand the impact and utility of OE. Multiple meta-analyses and retrospective studies have found no improvement of vision or survival with exenteration (6. 9-13). On the other hand, a few studies have found some improvement in survival after OE in patients with clinical features such as fever or rapidly progressing intracranial ROCM (14, 15). Our case comparison aligns with the former, that OE does not necessarily inform survival. Both cases 1 and 2 involve patients with multiple risk factors and severe ROCM extension that was eligible for OE. One patient (case 1) had full recovery despite deferring OE, while our other patient (case 2) unfortunately had continued systemic deterioration after OE leading to death. This highlights that mortality in ROCM is often a result of severe systemic disease in the setting of multiple comorbidities in addition to the severity of ROCM infection itself. When interpreting the mortality after OE, it is also important to consider that most patients who undergo OE have a more severe, extensive illness at presentation that contributes to their survival outcome. Although recovery and outcome may be uncertain, OE remains a critical step in the treatment of refractory cases of ROCM, in which potential benefits of controlling infection outweigh the risks and implications of OE.

There is currently no consensus on the standard of care for severe ROCM particularly when it comes to the decision to exenterate. Some hospitals have developed institution-specific scoring systems and treatment algorithms to evaluate the necessity of exenteration (3, 6–8). In their treatment algorithm, Kalin-Hajdu et al. identified that the extent of the loss of contrast enhancement, representing necrosed tissue, on orbital imaging can guide whether conservative debridement, transcutaneous retrobulbar antifungals, or OE is recommended (6). Transcutaneous retrobulbar amphotericin B (TRAMB) is a novel adjuvant therapy to systemic antifungals that has shown to significantly slow the progression of orbital disease in ROCM, improve vision, and decrease the need for exenteration (9–11, 20, 21). After including TRAMB in the aforementioned treatment algorithm, investigators found a decreased rate of exenteration with improved visual acuity and unchanged risk of mortality at their institution (9–11). Shakrawal et al. found that, among patients with diffuse orbital involvement of more than one quadrant and some preservation of vision, at least one TRAMB injection was sufficient in stabilizing the infection without the need for exenteration (21). In a retrospective cohort study, Karat et al. reported that cases with conservative management and TRAMB had a survival rate similar to patients who received OE as first-line treatment in other studies (22). However, ROCM may continue to progress despite amphotericin B injection as occurred in case 1 with infusion of amphotericin B, prompting the discussion of OE. A multicenter study identified that while TRAMB is associated with a decreased rate of OE in cases of ROCM with local orbital involvement, it did not influence OE rates in the setting of more extensive ROCM infection (23). Ultimately, adjuvant TRAMB may play a critical role in controlling and treating ROCM infection prior to considering exenteration and should continue to be incorporated in treatment algorithms.

The location of ROCM extension can also provide more guidance on the specifics of OE once appropriate. Mukit et al. demonstrated that in cases of posterior orbit infiltration, lid-sparing exenteration had a better functional and aesthetic outcome than conventional OE (24). In case 3, our patient recovered after a globe-sparing exenteration of medial orbit and underwent successful cosmetic reconstructions. This option for subtotal OE has yet to be further investigated and incorporated in ROCM treatment algorithms currently available in the literature. While modified OE is known to have better functional and cosmetic outcomes, more research is required to understand its efficacy in treating ROCM and the specific indications for subtotal over total OE (25). The localization of ROCM infection and ischemia and the reversibility of risk factors causing immunosuppression are likely critical factors in determining candidacy for subtotal OE. For the patient in case 3, the improvement of his AML, his primary immunosuppressive risk factor, and the localization of infection in the medial orbit were reassuring for a positive outcome. Considering these factors for survival outcome given his age and projected quality of life, globe-sparing intervention was advised. Meanwhile, patients in cases 1 and 2 had at least one unimproving risk factor and more rapid, diffuse spread of ROCM around the optic nerve and/or local structures. As a result, a conventional OE was advised. With further study establishing compared efficacy, future treatment algorithms can specify that in patients with localized orbital ROCM involvement on imaging and reversible or improving immunosuppressive risk factors, lid or globe-sparing OE is a viable option.

## 4 Conclusion

Management of comorbid risk factors, timely diagnosis of mucormycosis, and early and appropriate medical and/or surgical intervention are critical factors contributing to the functional and survival outcomes of patients with ROCM. Ideal exenteration timing and its impact on these outcomes remain uncertain. Each patient should undergo a multidisciplinary risk–benefit analysis to evaluate appropriateness of OE given its disfiguring and involved nature. Prior to considering any form of OE, adjuvant transcutaneous retrobulbar amphotericin B can slow orbital progression that has continued despite systemic antifungals and sinus debridements. Subtotal forms of OE, such as globe or lid-sparing OE, may be a viable alternative to conventional OE with less disfigurement and potential for vision preservation in the setting of reversible immunosuppression and focal ROCM involvement of the orbit.

## Data Availability

The datasets presented in this article are not readily available because of ethical and privacy restrictions. Requests to access the datasets should be directed to the corresponding author.
